# Determination of Sialic Acid Isomers from Released *N*-Glycans Using Ion Mobility Spectrometry

**DOI:** 10.1021/acs.analchem.2c00783

**Published:** 2022-09-19

**Authors:** Christian Manz, Montserrat Mancera-Arteu, Andreas Zappe, Emeline Hanozin, Lukasz Polewski, Estela Giménez, Victoria Sanz-Nebot, Kevin Pagel

**Affiliations:** † Department of Chemistry and Biochemistry, Freie Universität Berlin, Altensteinstr. 23A, 14195 Berlin, Germany; ‡ Department of Molecular Physics, Fritz Haber Institute of the Max Planck Society, Faradayweg 4-6, 14195 Berlin, Germany; § Department of Chemical Engineering and Analytical Chemistry, University of Barcelona, Martí i Franquès, 1-11, 08028 Barcelona, Spain

## Abstract

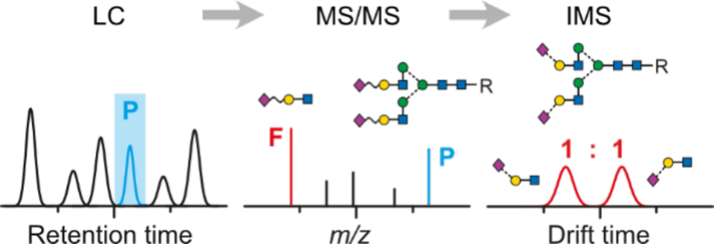

Complex carbohydrates are ubiquitous in nature and represent one of the major classes of biopolymers. They can exhibit highly diverse structures with multiple branched sites as well as a complex regio- and stereochemistry. A common way to analytically address this complexity is liquid chromatography (LC) in combination with mass spectrometry (MS). However, MS-based detection often does not provide sufficient information to distinguish glycan isomers. Ion mobility-mass spectrometry (IM-MS)—a technique that separates ions based on their size, charge, and shape—has recently shown great potential to solve this problem by identifying characteristic isomeric glycan features such as the sialylation and fucosylation pattern. However, while both LC-MS and IM-MS have clearly proven their individual capabilities for glycan analysis, attempts to combine both methods into a consistent workflow are lacking. Here, we close this gap and combine hydrophilic interaction liquid chromatography (HILIC) with IM-MS to analyze the glycan structures released from human alpha-1-acid glycoprotein (hAGP). HILIC separates the crude mixture of highly sialylated multi-antennary glycans, MS provides information on glycan composition, and IMS is used to distinguish and quantify α2,6- and α2,3-linked sialic acid isomers based on characteristic fragments. Further, the technique can support the assignment of antenna fucosylation. This feature mapping can confidently assign glycan isomers with multiple sialic acids within one LC-IM-MS run and is fully compatible with existing workflows for *N*-glycan analysis.

## Introduction

Glycosylation is a common post translational modification found on proteins and very important for their stability, activity, and function.^1^ As glycan biosynthesis is not template-driven, the nature of glycosylation may be different for each individual protein. In particular, glycosylation is very sensitive to the environment of the protein and changes are therefore often directly associated to diseases.^1,2^ In the case of *N*-linked glycans for example, pathologic changes can translate into altered levels of sialylation^3^ or fucosylation.^4^

The sialylation pattern is described by the type and linkage of sialic acid, which is a generic term for a family of more than 50 different acidic monosaccharides and is used synonymously in humans for its most prominent member, *N*-acetylneuraminic acid (Neu5Ac). Neu5Ac is usually found at the non-reducing end of branched *N*-glycans as a terminal monosaccharide residue connected to a galactose *via* α2,3- or α2,6-linkages. Due to its exposed location, sialic acids often participate as recognition sites in biological processes including cancerogenesis.^5^ As the up- or downregulation of each linkage isomer can correlate to different types of cancer,^6−8^ it is important to monitor the sialic acid linkage type and the glycan structure in general.

The detailed analysis of sialylated glycans is a very challenging task, especially when isomers have to be identified. Today, the gold standard method for glycan analysis relies on LC-MS and involves the enzymatic release of the glycans from a glycoprotein and derivatization with a fluorescence tag. While LC-MS is able to separate and identify most of the common *N-*glycan structures, a detailed assignment of α2,3- or α2,6-linkage isomers is still laborious and tedious due to their very similar MS/MS fragmentation patterns.^9,10^ In addition to conventional LC-MS, sequential digestion with several exoglycosidases can be applied to identify the regiochemistry of the sialic acid linkage.^11,12^ This however also leads to a significant increase in costs and analysis time. Recently, linkage-specific derivatization of α2,3- and α2,6-isomers in combination with matrix-assisted laser desorption/ionization-MS (MALDI-MS) emerged as a promising alternative to the slower LC-MS and enzyme-based approaches.^13,14^ Using this approach, the linkage types can be directly identified by a mass difference; however, the sample preparation is complex and not yet applicable in routine work.

As an alternative to common MS-based approaches, IMS recently emerged as a promising tool for glycomics.^15−17^ In IMS, ions travel through a gas-filled drift cell guided by an electrical field and are separated based on their charge, size, and shape.^18^ Although IMS is only able to partially resolve intact sialylated glycans,^19,20^ α2,3- and α2,6-sialic acid linkages can be unambiguously identified using a fragment-based approach.^21,22^ Furthermore, and in contrast to MALDI-MS, IMS can be easily implemented in existing LC-MS workflows and is fully compatible with commonly applied fluorescent labels.^23^

In this study, we assessed the potential of IM-MS to assign sialic acid linkage isomers on the level of released complex *N*-glycans. For this purpose, we selected human alpha-1-acid glycoprotein (hAGP) as a model due to its large *N*-glycan microheterogeneity and its potential as a biomarker in pancreatic cancer and other diseases. In addition, characterization of the sialic acid linkage type of hAGP *N*-glycans has been previously addressed by several LC-MS approaches,^10,12^ which allows a thorough validation of our results. Our data indicate that a direct injection IM-MS approach is able to identify the general sialic acid distribution of hAGP without prior derivatization. It allows the quantification of sialic acid linkage isomers individually for each isomeric class and is highly suitable for a rapid screening of glycoproteins. Subsequently, we used HILIC directly coupled to IM-MS to simultaneously analyze the glycan composition and sialylation pattern of complex *N*-glycans released from hAGP. The combination of both techniques proved to be a powerful tool for the characterization of *N*-glycans. It is able to fully resolve all sialic acid linkage isomers for each glycan individually while being fully compatible with existing workflows for *N*-glycan analysis.

## Materials and Methods

### Chemicals

All chemicals and reagents were at least analytical reagent grade and used without further purification. Rapid PNGase F and Rapid PNGase F Buffer were supplied by New England Biolabs (Ipswich, USA). Human alpha-1-acid glycoprotein (hAGP, ≥95%), Discovery Glycan solid phase extraction (SPE) tubes, TFA (≥99%), procainamide hydrochlorid (≥98%), and both trisaccharide standards 6′/3′-Sialyl-*N*-acetyllactosamine were purchased from Sigma-Aldrich (St. Louis, USA). The fucosylated standard 3′-Sialyl-Lewis X was supplied by Biosynth Carbosynth (UK). Hypercarb SPE tubes were purchased from Thermo Fisher Scientific (Waltham, USA). Ammonium formate (>99%) was obtained from VWR International (Radnor, USA). All solvents (acetonitrile, methanol, and water) were LC-MS grade and purchased from Sigma-Aldrich (St. Louis, USA).

### Sample Preparation for Native Glycans

Glycoprotein stock solution (10 μL, 10 mg/mL in water) was mixed with 6 μL of water and 4 μL of Rapid PNGase F Buffer and denatured at 95 °C for 10 min. After cooling to room temperature, 1 μL of Rapid PNGase F was added to the mixture and incubated at 50 °C for 10 min. Afterward, the released glycans were enriched with Hypercarb SPE tubes according to the vendor’s instruction, dried *via* SpeedVac (Thermo Fisher Scientific, Waltham, USA), and suspended in 50 μL of water:methanol:formic acid (1:1:0.1 v/v/%) prior to direct injection IM-MS analysis.

### Sample Preparation for Labeled Glycans

Glycoprotein stock solution (10 μL, 10 mg/mL in water) was mixed with 6 μL of water and 4 μL of Rapid PNGase F Buffer and denatured at 95 °C for 10 min. After cooling to room temperature, 1 μL of Rapid PNGase F was added to the mixture and incubated at 50 °C for 10 min. Afterward, the released glycans were labeled with procainamide according to established protocols.^23,24^ The labeled glycans were purified with the Discovery Glycan SPE tubes according to the vendor’s instruction, dried *via* SpeedVac (Thermo Fisher Scientific, Waltham, USA), and further suspended in 50 μL of water before storing them in an HPLC autosampler at 4 °C.

### Offline IM-MS Experiments

Traveling wave (TW) IM-MS measurements were performed on a Synapt G2-S HDMS instrument (Waters Corporation, Manchester, UK), described in detail elsewhere.^25^ Direct infusion measurements with released native glycans were performed in positive ion mode using platinum/palladium (Pt/Pd, 80/20) coated borosilicate capillaries prepared in-house. For nanoelectrospray ionization (nESI), typically 5 μL of the sample was loaded to a capillary and electrosprayed by applying a capillary voltage of 0.6–1.1 kV.

### Online HILIC-IM-MS Experiments

HPLC experiments were performed on a Acquity UPLC (Waters, Milford, USA) equipped with an autosampler, column oven, and a binary pump system. Released and procainamide-labeled glycans were separated by a glycan BEH amide column (150 mm × 2.1 mm, 130A, 1.7 μm, Waters, Milford, USA) before ESI ionization. Solvent A was 50 mM ammonium formate adjusted to pH 4.4, and solvent B was acetonitrile. The column temperature was set to 65 °C, and samples were analyzed at a flow rate of 0.4 mL/min using a linear gradient of 75–54% B from 0 to 35 min. The injection volume was 4–5 μL. The separated glycans were then ionized online with a capillary voltage of 2.2–2.5 kV.

Typical MS parameters in resolution mode (for offline and online measurements) for positive ion polarity were 30 V sampling cone voltage, 1 V source offset voltage, 120 °C source temperature, 0 V trap CE (MS), 27–30 V trap CE (MS/MS), 2 V transfer CE, and 3 mL/min trap gas flow. Ion mobility parameters were 5.0 V trap DC entrance voltage, 5.0 V trap DC bias voltage, −10.0 V trap DC voltage, 2.0 V trap DC exit voltage, −25.0 V IMS DC entrance voltage, 50–180 V helium cell DC voltage, −40.0 V helium exit voltage, 50–150 V IMS bias voltage, 0 V IMS DC exit voltage, 5.0 V transfer DC entrance voltage, 15.0 V transfer DC exit voltage, 150 m/s trap wave velocity, 1.0 V trap wave height voltage, 200 m/s transfer wave velocity, and 5.0 V transfer wave height voltage.

Data were acquired with MassLynx v4.1 and processed with Driftscope version 2.8 software (Waters, Manchester, UK) and OriginPro 8.5 (OriginLab Corporation, Northampton).

## Results and Discussion

### Direct Injection IM-MS Analysis of Released Glycans

As the separation power of IMS is often insufficient to fully separate larger glycan structures, intact precursors are usually cleaved into smaller fragments to deduce their overall structure from specific motifs. Such a characteristic fragment for the differentiation of sialic acid isomers *via* IM-MS was recently established on the level of glycopeptides. The proteolytic digestion of glycoproteins derived from Chinese hamster ovary cells (CHO) and human plasma resulted in *N*-glycopeptides, which were subsequently analyzed in a fragment-based IM-MS approach.^21,22^ The fragmentation of sialylated glycopeptides *via* collision-induced dissociation (CID) generates a characteristic B_3_ trisaccharide fragment, which contains a terminal α2,6- or α2,3-linked Neu5Ac. Both trisaccharide fragments exhibit almost baseline separated IMS features and can therefore be used to qualitatively differentiate sialic acid isomers on the level of glycopeptides. Although promising for estimating the overall isomer ratio, this approach is limited by the complexity of glycopeptide isomers and therefore struggles to elucidate the exact structure and sialic acid ratio of individual glycans.

**Figure 1:**
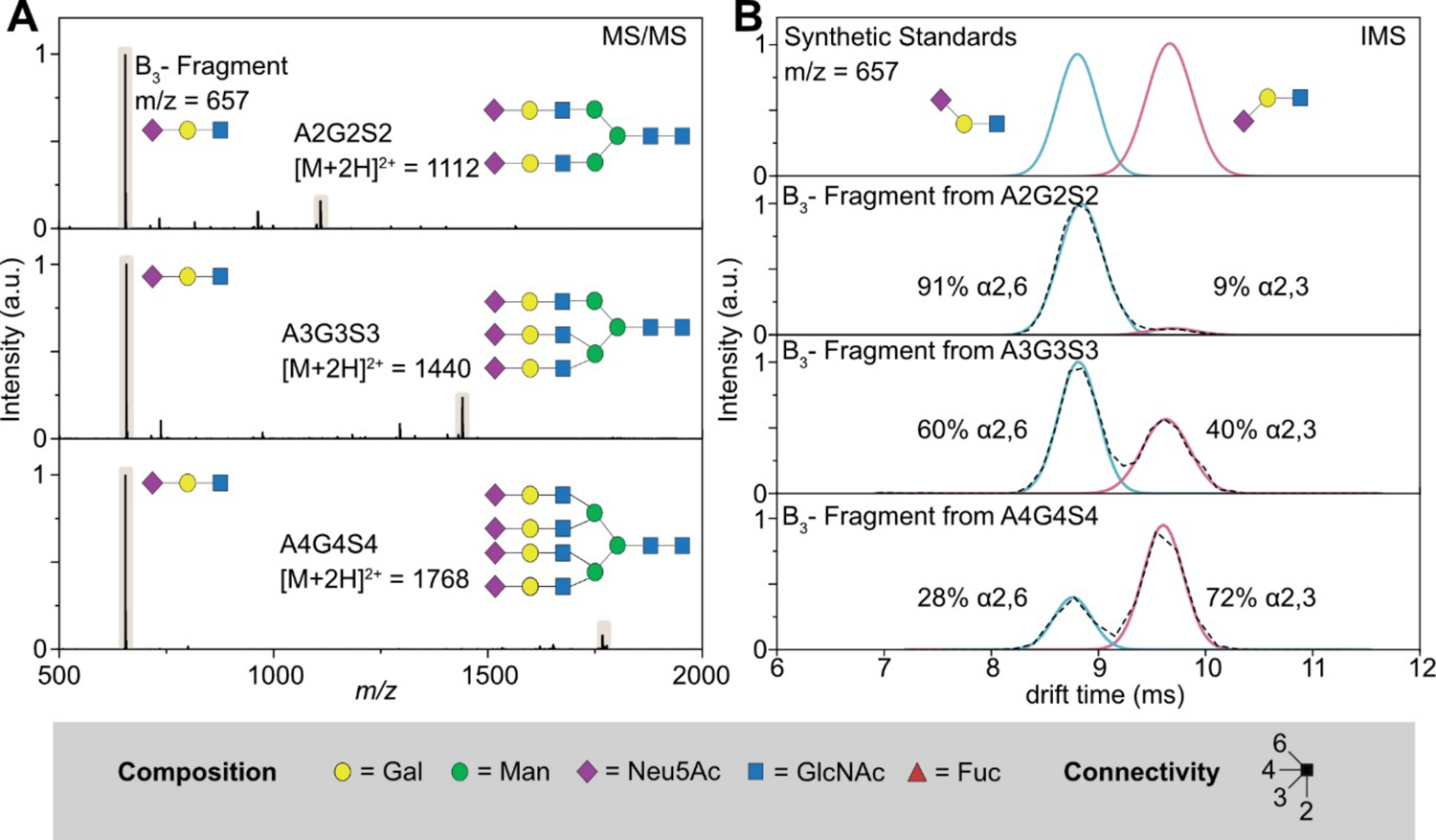
Differentiation of *N*-acetylneuraminic acid (Neu5Ac) linkage isomers using CID fragmentation and subsequent IM-MS analysis in positive ion mode. (A) MS/MS spectra of sialylated *N*-glycans A2G2S2 (top panel), A3G3S3 (middle panel), and A4G4S4 (bottom panel). Upon CID activation, each sialylated precursor exhibits a characteristic B_3_ trisaccharide fragment (*m*/*z* 657). (B) Mobilogram of two synthetic trisaccharide standards, which contain a terminal α2,6-linked sialic acid (blue trace) or a terminal α2,3-linked sialic acid (red trace). They can be used as reference to identify the isoforms of the B_3_ fragments cleaved from the sialylated glycan precursors. The black dotted line is the original ATD, while the blue and red traces represent the Gaussian fits to indicate α2,6- and α2,3-linked sialic acid isomers. Glycans are represented using the SNFG nomenclature, which depicts monosaccharides as colored symbols.^30^ The here crucial regiochemistry is defined by the angle of the glycosidic bond.

Therefore, we established the direct injection approach for complex *N*-glycan samples with the released glycans of hAGP as references. hAGP is an acute phase protein (APP) found in human plasma and displays a high *N*-glycan content (45%, w/w). The *N*-glycans of hAGP are relatively large (up to tetraantennary) and heavily sialylated, with both α2,6- and α2,3-linked Neu5Ac isomers.^10,26^ APPs in general are prone to contain potential biomarkers as they show changes on the protein and glycosylation level when confronted with inflammatory processes.^27,28^ In the case of hAGP, altered glycosylation was observed for several cancer types in which specific sialylated epitopes are formed.^29^ To release the glycans from the glycoprotein, hAGP was treated with PNGase F and the free glycans were enriched *via* PGC SPE. Subsequent direct injection MS analysis of the released glycans in positive ion mode reveals multiple highly sialylated glycan structures (see Table S1 in the Supporting Information). The fragmentation of these sialylated glycans *via* CID generates highly abundant B_3_ fragment ions (*m*/*z* = 657), which is exemplarily shown for the glycan species A2G2S2, A3G3S3, and A4G4S4 of hAGP in Figure 1. They represent typical complex *N*-glycans with multiple sialic acids, which are usually very demanding to distinguish by MS and MS/MS alone. The resulting MS/MS spectra reveal several fragment ions; in all cases, the characteristic B_3_ fragment (*m*/*z* = 657) was the dominant and most intense signal (Figure 1A). The arrival time distributions (ATDs) of the B_3_ fragments reveal two almost baseline separated features at ∼8.7 and ∼9.5 ms (Figure 1B). The structural assignment of both ATDs can be accomplished by comparison with two synthetic trisaccharide standards (Figure 1B, top panel). 6′-Sialyl-*N*-acetyllactosamine (blue trace) and 3′-sialyl-*N*-acetyllactosamine (red trace) share the same structure as the characteristic B_3_ fragment (*m*/*z* = 657) cleaved from sialylated *N*-glycans.^21,22^

The ATDs clearly reveal that the ratio of sialic acid isomers is highly dependent on the size of the glycan and the degree of branching (Figure 1B). While biantennary species almost exclusively contain α2,6-linked sialic acid (∼91% α2,6: 9% α2,3), a more balanced ratio of both isomers (∼60:40%) is observed for the triantennary species. The tetraantennary structure, on the other hand, exhibits a reversed trend with a higher content of α2,3-linked sialic acid (∼28:72%). Similar trends were reported based on LC-IM-MS data obtained from glycopeptides.^22^ Therefore, our results suggest that sialic acid isomers can be described qualitatively and quantitatively on the level of non-derivatized, native glycans in a direct injection IM-MS approach even for large glycan structures containing multiple sialic acids.

Although acidic glycans are usually analyzed in negative ion mode (due to the negative charge of sialic acids), this approach can only be performed in positive ion mode. Both ion polarities were tested by direct injection, and isomer separation was only observed in positive mode. Furthermore, only protonated precursors are amenable to the presented IMS analysis as metal adducted species (e.g., sodium adducts) do not allow us to distinguish the characteristic B_3_ fragment *via* IMS. The direct injection analysis of the complex *N*-glycans released from hAGP shows that the IM-MS workflow is suitable to distinguish sialic acid isomers qualitatively on the level of released glycans.

**Figure 2:**
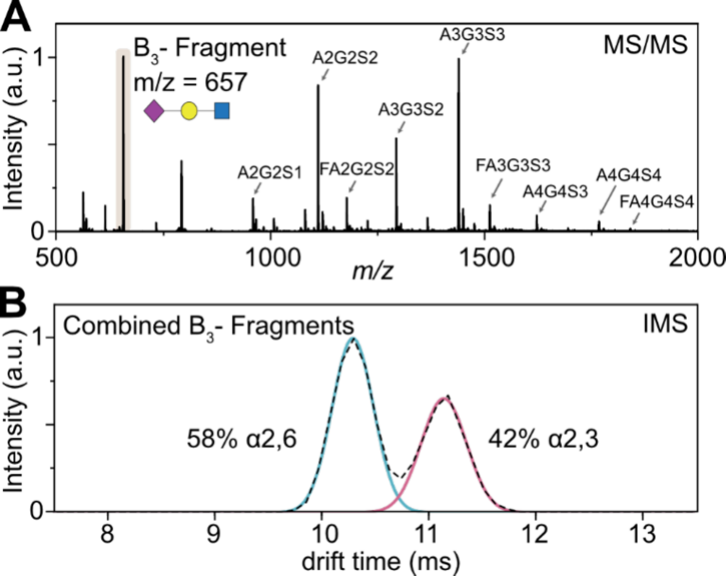
Non-targeted IM-MS analysis of released glycans from hAGP. (A) MS of native glycans shows nine complex-type, sialylated species. Without prior quadrupole isolation, CID activation leads to the fragmentation of all ionized glycans. The resulting B_3_ fragment results from all sialylated glycans. (B) The ATD of the B_3_ fragment represents the averaged drift time of α2,6- and α2,3-linked sialic acids for all sialylated glycans and can be used to estimate the overall sialic acid ratio.

In addition to the targeted approach described for individual glycans, we assessed the possibility to quantify the sialylation by IMS in a non-targeted approach. For this, we induced fragmentation of all precursor ions without prior mass selection in the quadrupole, which results in the combined ATD from all released *N*-glycans (Figure 2). The overall ratio of sialic acid linkage isomers released from hAGP shows a balanced ratio of 58% α2,6- vs 42% α2,3-linked sialic acids. This ratio matches values obtained in earlier IMS experiments on glycopeptides.^22^ This underlines the quantitative character of the presented fragment-based IMS approach on the level of released glycans.

Taken together, the results show the great potential of direct infusion nESI as a high throughput approach to recognize changes in the sialic acid isomer ratio without prior derivatization steps. IMS can quantitatively detect minor isomeric components with relative concentrations as low as 1%.^15^ The method described here is therefore well suited to quantitatively describe the ratio of α2,6- and α2,3-linked Neu5Ac isomers without chromatographic separation. In comparison to the corresponding glycopeptide workflows,^21,22,31^ it is more straightforward to identify sialic acid isomers on the level of released glycans. Sialic acid isomers of complex *N*-glycans (like hAGP) can be identified and quantified separately for individual *N*-glycan classes as bi-, tri-, and tetraantennary glycans. In the case of glycopeptides, this is much more challenging as not only the microheterogeneity of the glycans (i.e., multiple possible isomers on one glycosylation site) but also the macroheterogeneity of the glycopeptides (i.e., multiple possible glycosylation sites) needs to be considered. The presented approach is therefore a major advancement for screening purposes in clinical biomarker research where general statements about sialic acid linkage isomers are required to identify pathological changes.

### HILIC-MS Characterization of *N*-Glycans Released from hAGP

When hyphenated to liquid chromatography, the above-described method should in principle be able to provide glycan-resolved sialylation data. To test this, we implemented the developed IMS technique into the existing gold standard HILIC-MS *N*-glycan analysis workflow. Accordingly, the sample preparation for hAGP was modified to match typical HILIC-FLD and HILIC-MS workflows: the *N*-glycans were cleaved from the glycoprotein via PNGase F digestion and directly labeled at the free reducing end with procainamide. As the LC-MS and also the LC-IM-MS workflow are not dependent on the utilized fluorescent dye, this method is generally applicable to any available reducing end modification. Subsequently, the labeled glycans were purified using HILIC SPE and analyzed by HILIC-ESI-MS. More than 20 individual glycan species were identified based on their retention time, mass, and literature data^12^ (see Table S2 in the Supporting Information). According to the elution order of the identified glycans, the chromatogram can be divided into three areas, corresponding to bi-, tri-, and tetraantennary glycans. Biantennary glycans elute first (less than ∼20 min) followed by triantennary (from 20 to 26 min) and tetraantennary species (greater than ∼26 min). Although HILIC is able to separate multiple glycan isomers, it struggles to confidently identify all structural components. Especially, the orientation of the terminal sialic acid building blocks can often only be identified on the basis of glucose units (GU).^32^ GU values serve as reference standards to calibrate relative retention times of each eluting species, which can further be compared with database information.^33^ However, with growing complexity of glycans, the LC resolving power can reach its limit and database assignments may be inconclusive. Especially, unknown samples are challenging to characterize solely on the basis of LC-MS data and GU databases. Therefore, additional experiments are usually required to confidently assign all structural elements.

### Quantitative Assignment of Sialic Acid Isomers Based on LC-IM-MS

The incorporation of IMS into the described standard HILIC-MS workflow is straightforward and does not require changes to the general routine. The application of HILIC-IM-MS for complex *N*-glycans and glycopeptides was shown in prior studies and showed promising results for the differentiation of branching isomers.^34^ The application for highly sialylated and fucosylated glycans, however, still needs to be shown and represents a current analytical challenge in the field of glycomics. We therefore applied HLIC-IM-MS in a data-dependent acquisition on the released glycans of hAGP to qualitatively and quantitatively describe all sialylated species.

**Figure 3:**
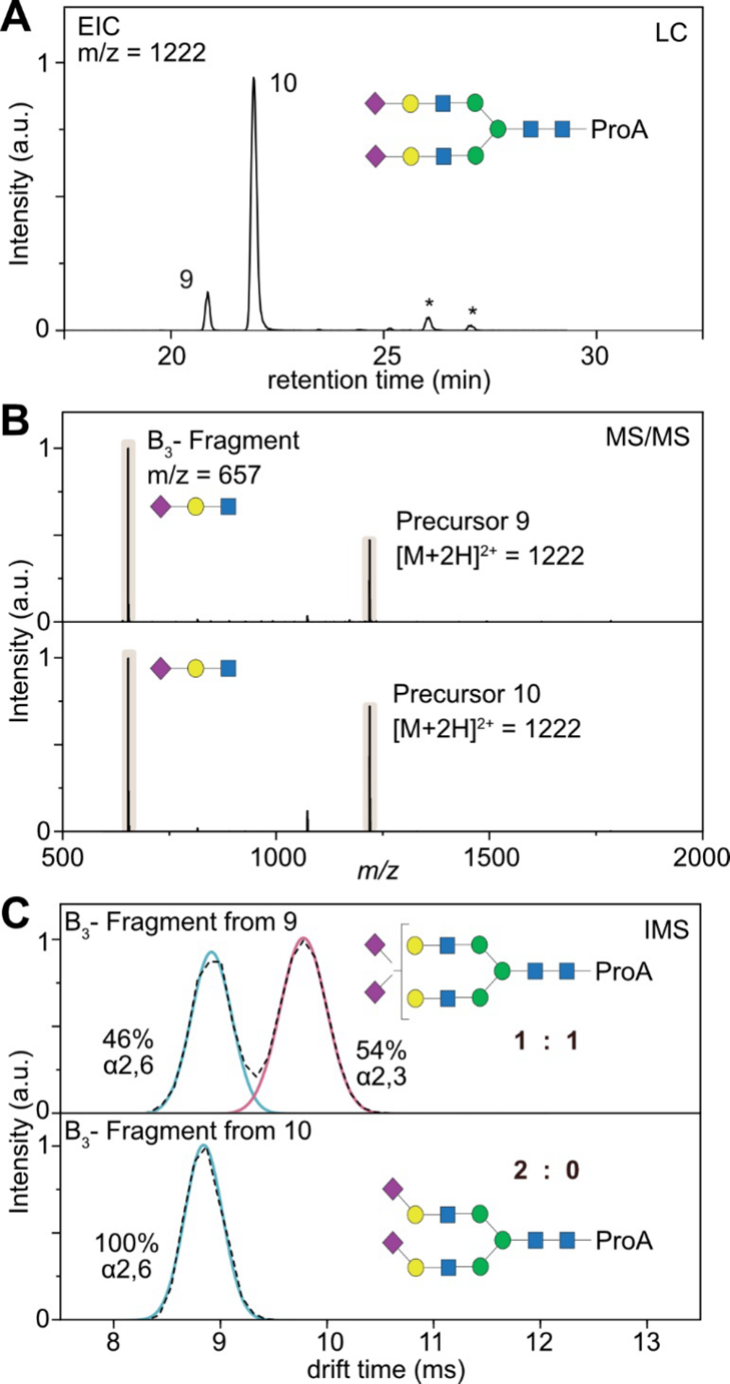
HILIC-CID-IM-MS feature mapping of released glycans of hAGP. (A) Extracted ion chromatogram (EIC) of a doubly sialylated biantennary glycan (*m*/*z* = 1222) in positive ion mode. Minor peaks marked with an asterisk are fragment ions generated from larger glycans. (B) MS/MS spectra of the precursor ions 9 and 10, which are almost identical and show the dominant B_3_ trisaccharide fragment. (C) Mobilograms of the B_3_ fragment generated from precursor ions 9 and 10. Comparison with the synthetic standards (red and blue overlay) allows us to identify the sialic acid isoforms and to deduce the general structure of the glycans 9 and 10. The numbering of the LC peaks refers to Tables S2 and S4 in the Supporting Information, which contains all observed glycans.

The general workflow is shown in Figure 3 for the doubly sialylated biantennary species (A2G2S2). This glycan structure shows two well-separated peaks in the HILIC chromatogram (Figure 3A) but exhibits identical MS/MS spectra in positive ion mode (Figure 3B) as both species correspond to isomeric structures. To investigate if these isomers differ in the orientation of the terminal sialic acid residues, the mobilograms of the B_3_ fragments obtained from both precursors were studied. As shown in Figure 3C, they significantly differ from each other. The fragment generated from peak 9 shows two features in the mobilogram with similar peak areas (46:54%), while the fragment of peak 10 only shows a single feature in the mobilogram (100%). The numbering of the LC peaks refers to Tables S2 and S4 in the Supporting Information and contains all observed glycans. In contrast to the direct injection experiments shown before, the upstream separation of isomers achieved by HILIC in combination with the IMS peak areas and drift times enables a quantitative assessment of sialic acid isomer proportions. In the case of A2G2S2, only three possible sialic acid ratios are possible (2× α2,6; 2× α2,3; or a 1:1 mix of both). The proportions derived from IMS separation allow a confident and simple structural assignment of both LC peaks. While the glycan corresponding to LC peak 9 contains a mixture of one α2,6- and one α2,3-linked sialic acid, the glycan corresponding to LC peak 10 exclusively contains α2,6-linked sialic acids located at both terminal positions of the biantennary structure. This structural assignment shows that the retention time of a glycan correlates with the type of sialic acid linkage. The higher the content of α2,6-linked isomers, the later the glycan species will elute, which is in good agreement with reported HILIC data.^10^ The structural assignments are confirmed by previous assignments of hAGP *N*-glycans using exoglycosidase digestions^12^ and MS/MS^10^ demonstrating the reliability of the established LC-IM-MS method.

As α2,6- and α2,3-linked sialic acid residues have different stabilities in the gas phase,^22^ the collision energy plays an important role in the quantitative assessment of the sialic acid linkage ratio. Therefore, we tested the stability of the observed B_3_ fragment under various activation energies with a biantennary glycan with two sialic acid residues as reference (see Table S3 in the Supporting Information). In contrast to previous studies on glycopeptides,^22^ we observe a much wider window of stability on the level of released glycans (collision energy from 20 up to 40 V) in which it was possible to assess the sialic acid linkage ratio. The most accurate results, however, were obtained for activation energies between 27 and 30 V; therefore, this collision energy was used throughout this study.

Although the total number and proportion of sialic acid isomers can be identified based on the presented data, no information on the relative position of the sialic acids on the individual antennae is obtained. HILIC columns are described for their potential to separate glycan isomers that differ in the linkage-type of terminal monosaccharides such as sialic acids.^35,36^ However, branching isomers with the same sialic acid linkage-type seem to be unresolved by HILIC chromatography, probably due to the equal hydrophilicity of these isomers. Recently, sialic acid derivatization in combination with reversed-phase LC separation showed great potential for this purpose.^37^ For an application in routine diagnostics, however, these details are usually not important. Instead, a general quantitative and qualitative assignment is more crucial to monitor pathological changes.

**Figure 4:**
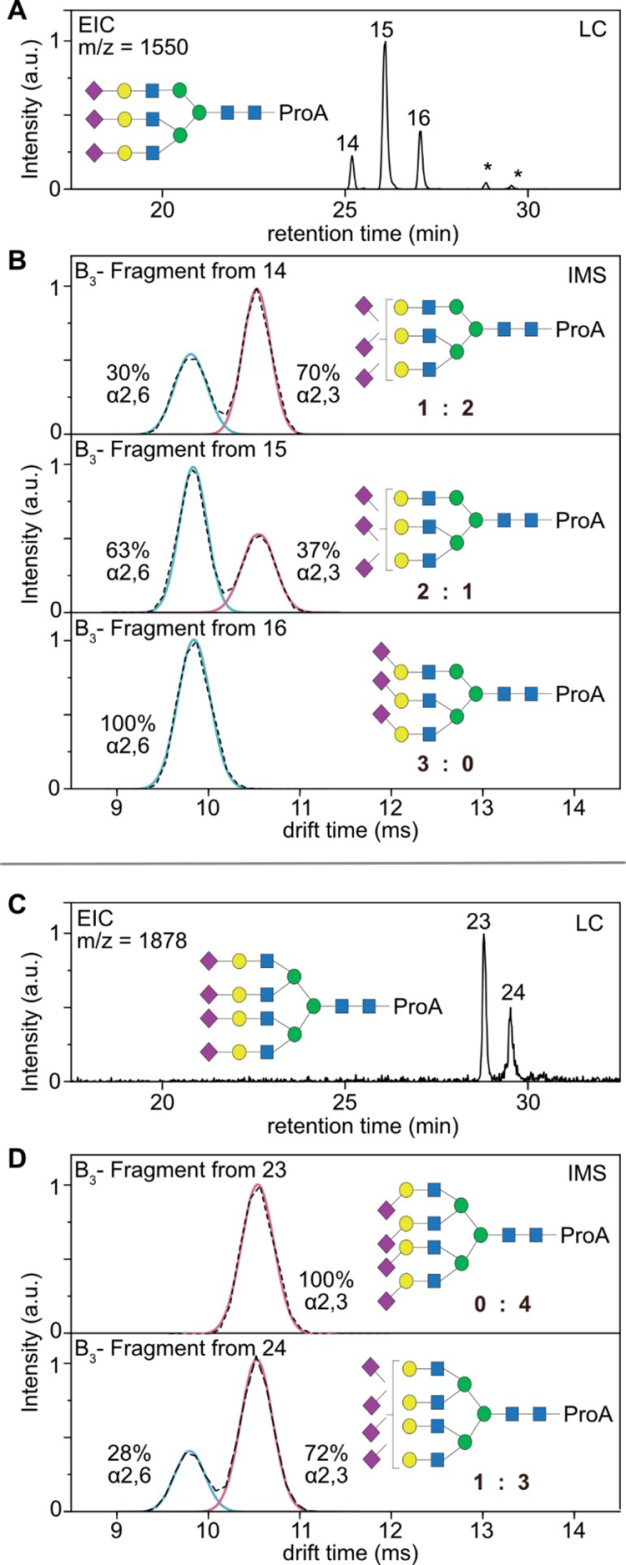
HILIC-IM-MS analysis of large, sialylated glycans. (A) Extracted ion chromatogram (EIC) of a triply sialylated triantennary glycan (*m*/*z* = 1550) of hAGP in positive ion mode. Minor peaks marked with an asterisk are fragment ions generated from larger glycans. (B) Mobilograms of the B_3_ fragment generated from precursor ions 14–16. (C) Extracted ion chromatogram (EIC) of a quadruply sialylated tetraantennary glycan (*m*/*z* = 1878) of hAGP in positive ion mode. (D) Mobilograms of the B_3_ fragment generated from precursor ions 23 and 24. Comparison with the synthetic standards (red and blue overlay) allows us to identify the sialic acid isoforms and to deduce the general structure of the glycan species.

Similarly to the biantennary glycans, the LC-IM-MS workflow can also be applied to larger sialylated structures. Figure 4 shows the analysis of the fully sialylated tri- and tetraantennary glycans released from hAGP. The EIC of *m*/*z* = 1550 corresponds to a triply sialylated, triantennary glycan and shows three distinct LC peaks (Figure 4A). As shown in Figure 4B, the mobilogram of the B_3_ fragment generated from LC peak 14 reveals a peak area ratio of 1:2 (30% vs 70%), which indicates that the three antennae in total contain one α2,6- and two α2,3-linked sialic acids. The fragment of LC peak 15 has a ratio of 2:1 (63% vs 37%), which indicates the presence of two α2,6-linked sialic acids and one α2,3-linked sialic acid. The latest eluting LC peak 16 exclusively contains α2,6-linked sialic acid. The quadruply sialylated tetraantennary species show two signals in the chromatogram (LC peaks 23 and 24; Figure 4C). The ATD reveals that isomer 23 exclusively contains α2,3-linked sialic acid, while isomer 24 shows a 1:3 ratio of α2,6:α2,3-linked sialic acid (Figure 4D). These quantitative sialic acid assignments are in good agreement with the general ratios obtained by the direct injection approach (Figure 1B). After chromatographic separation, however, only integer ratios of sialic acid isomers are possible, which significantly simplifies the detailed assignment of larger glycan structures. The determined collision energy window (Table S3 in the Supporting Information) seems to be valid for all sizes of sialylated glycans up to tetraantennary species, but this value might be different for other structures. It is therefore important to tightly control the collision energy in quantitative experiments and possibly reevaluate this fragmentation window for very small or very large sialylated glycans (e.g., *O*-glycans or poly-LacNAc structures). A comprehensive list of all identified glycans from hAGP and their respective sialic acid composition is shown in the Supporting Information (see Tables S2 and S4).

Taken together, the presented results show the universal applicability of the approach to all sialylated *N*-glycans independent of their size and structure. In addition to the diagnostic nature of the B_3_ fragment to distinguish α2,6- and α2,3-linked sialic acid isomers, the workflow can be used to derive quantitative information based on the relative IMS peak area of the two isomers, thereby benefiting the general structural assignment of sialylated glycans.

### Assignment of Fucosylated Complex *N*-Glycans

Another important structural motif of *N*-glycosylation is the type and level of fucosylation. Similar to the sialylation pattern, fucosylation can be used as a potential biomarker for cancer and therefore represents a particular important target for diagnostics applications. Fucosylation of complex glycans frequently occurs as core fucosylation linked *via* α1,6 to the *N*-acetyl-glucosamine at the reducing end. However, it can also be present as antenna fucosylation, which mainly occurs *via* α1,3-linkage at the antenna *N*-acetylhexosamine but can occasionally be linked to galactose residues to form typical blood group antigens.^23,38^ For diagnostic purposes, the primary concern is to distinguish between core and antenna fucosylation, while the secondary concern is related to the actual fucosylation motif (blood group antigens).

**Figure 5:**
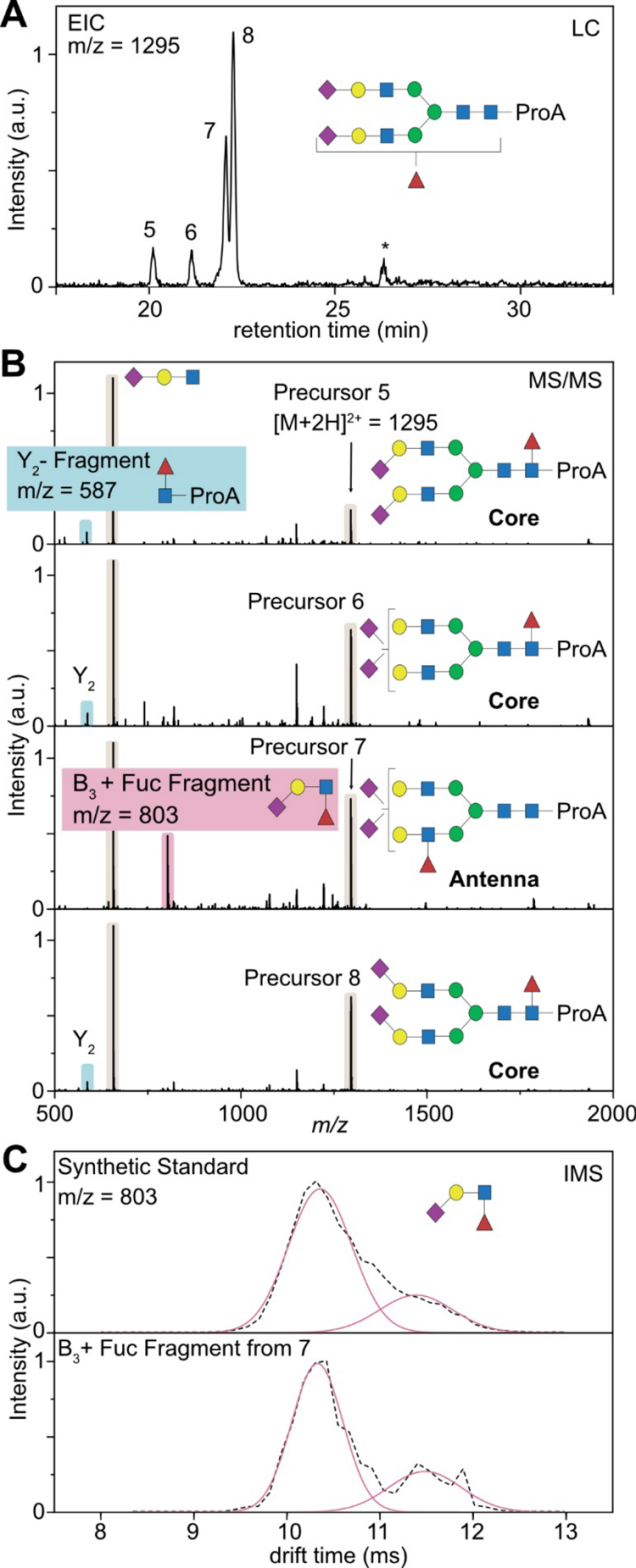
Determination of the fucosylation pattern based on HILIC-IM-MS. (A) Extracted ion chromatogram (EIC) of a doubly sialylated biantennary glycan with one fucose attached (*m*/*z* = 1295). Minor peaks marked with an asterisk are fragment ions generated from larger glycans. (B) MS/MS spectra of the precursor ions 5–8, which are almost identical and show the dominant B_3_ trisaccharide fragment. One major difference stems from either Y_2_ fragmentation (highlighted in blue) or B_3_ fragmentation (highlighted in red). (C) Mobilograms of the B_3_ + fucose fragment generated from precursor ion 7. Comparison with a synthetic standard (3′-Sialyl-Lewis-X) allows us to identify the fucose isoforms and confirm the native state of the fucosylation.

In the HILIC-IM-MS studies, we further identified three fucosylated species, namely, FA2G2S2, FA3G3S3, and FA4G4S4. For the smallest observed fucosylated species FA2G2S2, we observe four distinct isomers for the mass of *m*/*z* = 1295, which represents the biantennary glycan with two sialic acids and one fucose attached (Figure 5A). The identification of sialic acid linkage isomers is based on the ATD of the B_3_ fragment for each separated species and reveals that precursor 5 has a 0:2 ratio, precursors 6 and 7 have a 1:1 ratio, and precursor 8 has a 2:0 ratio (see Table S4 in the Supporting Information). The trend in elution order is similar to that in non-fucosylated glycans and is highly dependent on the type of sialic acid attached. For isomers 6 and 7, however, we can observe two baseline separated peaks in the chromatogram, although both species contain a 1:1 ratio of α2,6- and α2,3-linked sialic acid isomers. Therefore, the differences in the retention times are likely resulting from a different fucosylation pattern (core vs antenna). A tentative identification of core or antennary fucosylation can be achieved by the analysis of the MS/MS data (Figure 5B). Isomers 5, 6, and 8 share a very similar fragmentation pattern and a common Y_2_ fragment (*m*/*z* = 587), which corresponds to [GlcNAc + fucose + procainamide + H]^+^. It was shown previously that this fragment is characteristic for core fucosylation.^39^ Fucose monosaccharides tend to migrate along the oligosaccharide backbone during mass spectrometry analysis.^40,41^ However, core fucose is usually strongly bound to the sugar core^42^ and the migration reaction is known to be inhibited by immobilization of the charge at the procainamide label.^43^ Therefore, this specific fragment is a strong indicator for core fucosylation in the case of derivatized glycans.

On the other hand, there is a clearly recognizable difference in the MS/MS spectra for isomer 7. Instead of a Y_2_ fragment indicating core fucosylation, isomer 7 shows an intense fragment signal at *m*/*z* = 803. This signal corresponds to a terminal B_3_ + fucose fragment [Neu5Ac + Gal + GlcNAc + Fuc + H]^+^. Although this fragment is indicative for fucosylation at the antenna, antennary fucosylation is very labile and requires low activation energies for migration reactions to occur.^40,42^ In this case, IMS might support the discrimination between native antennary fucosylation and non-native, migrated structures. Recent studies showed that natively fucosylated structures yield reproducible ATDs, which can be compared to suitable standards, while rearranged structures exhibit multiple features in the ATD.^44^ This can be explained by the fucose migration mechanism as the rearrangement might only occur to certain functional groups (such as *N*-acetylation on Neu5Ac and GlcNAc) and therefore creates distinguishable isomeric structures.

A typical antennary fucosylation of a sialylated species is the Sialyl Lewis X (α1,3-linked fucose) epitope.^45^ We therefore compared the ATD of the occurring terminal B_3_ + fucose fragment [Neu5Ac + Gal + GlcNAc + Fuc + H]^+^ with a commercially available standard of Sialyl Lewis X (Figure 5C). Both ATDs show one distinct feature at ∼10.5 ms and additionally have a small shoulder on the right side at 11.6 ms, which is in good agreement with literature values and supports the assignment as the native Sialyl Lewis X epitope.^44^ This observation is also in agreement with recent experiments conducted with sequential enzymatic reactions utilizing fucosidase digestions.^12^ As we did not observe species that correspond to fucose migration, we can only tentatively assign the fucose epitope.

Both larger fucosylated structures observed in this study (FA3G3S3 and FA4G4S4) were examined in the same way to identify both the sialic acid and the fucosylation pattern. The characterization of the sialic acid linkage isomer ratio was performed on the basis of the generated B_3_ fragment and allowed for unambiguous assignment of all sialylated isomers (see Tables S2 and S4 in the Supporting Information). We further checked the fragmentation pattern of all fucosylated species for either Y_2_ or B_3_ fragmentation to indicate core or antenna fucosylation (see Figures S1 and S2 in the Supporting Information). All tri- and tetraantennary species with one fucose showed exclusively B_3_ fragments, which is indicative for antennary fucosylation. In addition, we compared the ATD of all B_3_ fragments with that of the reference Sialyl Lewis X. As all ATDs showed good agreement with the ATD from Sialyl Lewis X, we assume that the triantennary and tetraantennary glycans of hAGP exclusively contain antenna fucosylation and more specifically Sialyl Lewis X epitopes.

The above examples show that LC-IM-MS can support the differentiation of core and antenna fucosylation for derivatized glycans. However, to fully explore the potential of HILIC-IM-MS for fucosylation analysis, more detailed experiments are needed in the future. The advantage of this workflow is the ease and speed of application as it can be performed within a single LC run without further modification of established LC-MS workflows.

## Conclusions

In conclusion, IMS has the potential to fill the informational gap in *N*-glycan analysis left by LC-MS. It enables the analyst to unambiguously identify characteristic structural motifs such as the sialylation pattern in both a qualitative and quantitative way. The regiochemistry of terminal sialic acid linkages can be identified based on B_3_-type fragments that are cleaved from the corresponding *N*-glycan before the IMS separation and further quantified based on their respective peak areas in the mobilogram. Meanwhile, in direct injection approaches, this allows us to derive a general α2,6:α2,3 ratio; LC separation in combination with IM-MS allows us to deduce more accurate *N*-glycan structures. Here, this workflow was applied to characterize the sialylation pattern of biantennary, triantennary, and tetraantennary glycans released from hAGP. In some cases, it is further possible to distinguish core and antennary fucosylation based on the LC-MS/MS data in conjunction with IMS data. The presented approach adequately complements existing LC-MS workflows and allows us to obtain information on structural motifs without the need for further sample preparation and instrument modification. Given the already broad distribution of commercial IM-MS instruments, the proposed fragment-based approach can be readily implemented and applied in many laboratories. The proposed workflow, however, is specifically designed for instruments that are able to isolate and fragment a precursor ion before IMS separation. Front-end IMS instruments might require further investigation to implement this fragment-based approach as experimental parameters such as fragmentation energy and required LC resolution might deviate from the suggested conditions. As IMS identification works independently from the LC dimension, the approach is not limited to HILIC methods and can be used with PGC, C18, or even capillary electrophoresis. Furthermore, the strategy does not require special sample preparation and can therefore be easily implemented into existing methods.

Very few other methods are able to identify a comparable level of structural information within the time frame of a single LC run. LC-IM-MS therefore has the potential to serve as a universally applicable analysis tool for future *N-*glycan analysis. In principle, the method should also be applicable for *O*-glycan analysis. It was for example shown that the IMS of characteristic terminal fragments can also be used to identify the fucosylated structural motifs of glycans,^38^ and further experiments are required to test if this approach is also compatible with existing LC-MS workflows.

## Supporting Information Available

Listing of all native *N*-glycans observed *via* direct injection IM-MS; additional data on glycan composition and sialic acid linkage ratio of all procainamide-labeled glycans observed *via* HILIC-IM-MS; additional data on activation energy required to fragment biantennary glycan A2G2S2 (PDF)
