# Supplementary material for: Determination of Sialic Acid Isomers from Released N-Glycans Using Ion Mobility Spectrometry
Source: Anal Chem. 2022 Sep 19;94(39):13323–31. doi: 10.1021/acs.analchem.2c00783 (PMC9535620; doi:10.1021/acs.analchem.2c00783)
Supplement: Supplementary file 1 — ac2c00783_si_001.pdf [file ac2c00783_si_001.pdf]

Supporting Information

Determination of Sialic Acid Isomers from Released *N*-glycans Using Ion Mobility Spectrometry

Christian Manz[1,2], Montserrat Mancera-Arteu[3], Andreas Zappe[1], Emeline Hanozin[1,2], Lukasz Polewski[1,2], Estela Giménez[3], Victoria Sanz-Nebot[3], Kevin Pagel[1,2,*]

1 Freie Universität Berlin, Department of Chemistry and Biochemistry, Altensteinstr. 23A, 14195 Berlin, Germany

2 Fritz Haber Institute of the Max Planck Society, Department of Molecular Physics, Faradayweg 4-6, 14195 Berlin, Germany

3 University of Barcelona, Department of Chemical Engineering and Analytical Chemistry, Martí i Franquès, 1-11, 08028 Barcelona, Spain

* Corresponding author. E-Mail: kevin.pagel@fu-berlin.de

Table of Content

Table S-1: Observed glycan species in the direct injection approach for the analysis of native, released glycans of hAGP in positive ion mode. S-2

Table S-2: Compositional analysis of released and procainamide-labeled N-glycans from hAGP based on LC-MS results. S-3

Table S-3: Stability analysis of B3 trisaccharide fragment cleaved from A2G2S2. ....S-4

Table S-4: LC-IM-MS analysis of procainamide-labeled N-glycans from hAGP. S-5

Figure S-1: Determination of fucosylation pattern for triantennary species. S-6

Figure S-2: Determination of fucosylation pattern for tetraantennary species. S-7

Table S-1: Observed glycan species in the direct injection approach for the analysis of native, released glycans of hAGP in positive ion mode.

Glycan species	*m/z*	z	m	Hexose	HexNAc	Fuc	Sia
A2G2S1	966.85	2	1931.7	5	4	0	1
A2G2S2	1112.39	2	2222.79	5	4	0	2
FA2G2S2	1185.43	2	2368.85	5	4	1	2
A3G3S2	1294.96	2	2587.92	6	5	0	2
A3G3S3	1440.51	2	2879.02	6	5	0	3
FA3G3S3	1513.54	2	3025.08	6	5	1	3
A4G4S3	1623.08	2	3244.15	7	6	0	3
A4G4S4	1768.63	2	3534.60	7	6	0	4
FA4G4S4	1841.65	2	3681.31	7	6	1	4

Table S-2: Compositional analysis of released and procainamide-labeled N-glycans from hAGP based on LC-MS results.

LC peak no.	Retention time	Glycan species	*m/z*	z	m	Hexose	HexNAc	Fuc	Sia
1	17.4	A2G2S1	1076.43	2	2150.86	5	4	0	1
2	17.6	A2G2S1	1076.43	2	2150.86	5	4	0	1
3	18.6	A2G2S1	1076.43	2	2150.86	5	4	0	1
4	18.7	A2G2S1	1076.43	2	2150.86	5	4	0	1
5	20.3	FA2G2S2	1294.99	2	2587.98	5	4	1	2
6	21.3	FA2G2S2	1294.99	2	2587.98	5	4	1	2
7	22.3	FA2G2S2	1294.99	2	2587.98	5	4	1	2
8	22.5	FA2G2S2	1294.99	2	2587.98	5	4	1	2
9	20.6	A2G2S2	1221.98	2	2441.96	5	4	0	2
10	21.7	A2G2S2	1221.98	2	2441.96	5	4	0	2
11	22.2	A3G3S2	1404.54	2	2807.08	6	5	0	2
12	23.2	A3G3S2	1404.54	2	2807.08	6	5	0	2
13	24.1	A3G3S2	1404.54	2	2807.08	6	5	0	2
14	24.7	A3G3S3	1550.07	2	3098.14	6	5	0	3
15	25.6	A3G3S3	1550.07	2	3098.14	6	5	0	3
16	26.5	A3G3S3	1550.07	2	3098.14	6	5	0	3
17	25.7	FA3G3S3	1623.08	2	3244.16	6	5	1	3
18	26.2	FA3G3S3	1623.08	2	3244.16	6	5	1	3
19	26.6	FA3G3S3	1623.08	2	3244.16	6	5	1	3
20	26.1	A4G4S3	1732.60	2	3463.2	7	6	0	3
21	27.0	A4G4S3	1732.60	2	3463.2	7	6	0	3
22	27.6	A4G4S3	1732.60	2	3463.2	7	6	0	3
23	28.2	A4G4S4	1878.12	2	3754.24	7	6	0	4
24	28.9	A4G4S4	1878.12	2	3754.24	7	6	0	4
25	28.9	FA4G4S4	1951.11	2	3900.22	7	6	1	4
26	29.3	FA4G4S4	1951.11	2	3900.22	7	6	1	4
27	29.7	FA4G4S4	1951.11	2	3900.22	7	6	1	4

Table S-3: Stability analysis of B3 trisaccharide fragment cleaved from A2G2S2. To assess a window of collision energy for the quantitative analysis of sialic acid residues, LC peak no. 9 (table S-2) was fragmented with various collision energies. Sialic acid linkage ratio is derived by the peak area of the characteristic B_3 fragment (m/z = 657) in percent. Afterwards this percentage ratio is normalized to the number of sialic acids (in this case two). According to previous studies, this isomer of A2G2S2 should contain a 1:1 mixture of α2,6 and α2,3 sialic acid residues.

Collision energy	Sialic acid ATD ratio	(rel. in %)	Sialic acid ratio	(norm. in %)
(in V)	α2,6	α2,3	α2,6	α2,3
20	35	65	0.7	1.3
25	41	59	0.8	1.2
27	46	54	0.9	1.1
30	54	46	1.1	0.9
35	59	41	1.2	0.8
40	64	36	1.3	0.7

Table S-4: LC-IM-MS analysis of procainamide-labeled N-glycans from hAGP. Sialic acid linkage ratio is derived by the peak area of the characteristic B_3 fragment (m/z = 657) in percent for each glycan species. Afterwards this percentage ratio is normalized to the number of sialic acids. This ratio is then rounded to absolute integer values.

LC peak.	Glycan	Sia ATD ratio	(rel. in %)	Ratio	(norm. in %)	Ratio	(abs.)
		α2,6	α2,3	α2,6	α2,3	α2,6	α2,3
1	A2G2S1	0	100	0,0	1,0	0	1
2	A2G2S1	0	100	0,0	1,0	0	1
3	A2G2S1	100	0	1,0	0,0	1	0
4	A2G2S1	100	0	1,0	0,0	1	0
5	FA2G2S2	0	100	0,0	2,0	0	2
6	FA2G2S2	46	54	0,9	1,1	1	1
7	FA2G2S2	54	46	1,1	0,9	1	1
8	FA2G2S2	100	0	2,0	0,0	2	0
9	A2G2S2	46	54	0,9	1,1	1	1
10	A2G2S2	100	0	2,0	0,0	2	0
11	A3G3S2	0	100	0,0	2,0	0	2
12	A3G3S2	43	57	0,9	1,1	1	1
13	A3G3S2	100	0	2,0	0,0	2	0
14	A3G3S3	30	70	0,9	2,1	1	2
15	A3G3S3	63	37	1,9	1,1	2	1
16	A3G3S3	100	0	3,0	0,0	3	0
17	FA3G3S3	36	64	1,1	1,9	1	2
18	FA3G3S3	59	41	1,8	1,2	2	1
19	FA3G3S3	90	10	2,7	0,3	3	0
20	A4G4S3	0	100	0,0	3,0	0	3
21	A4G4S3	40	60	1,2	1,8	1	2
22	A4G4S3	83	17	2,5	0,5	3	0
23	A4G4S4	0	100	0,0	4,0	0	4
24	A4G4S4	28	72	1,1	2,9	1	3
25	FA4G4S4	11	89	0,4	3,6	0	4
26	FA4G4S4	24	76	1,0	3,0	1	3
27	FA4G4S4	47	53	1,9	2,1	2	2

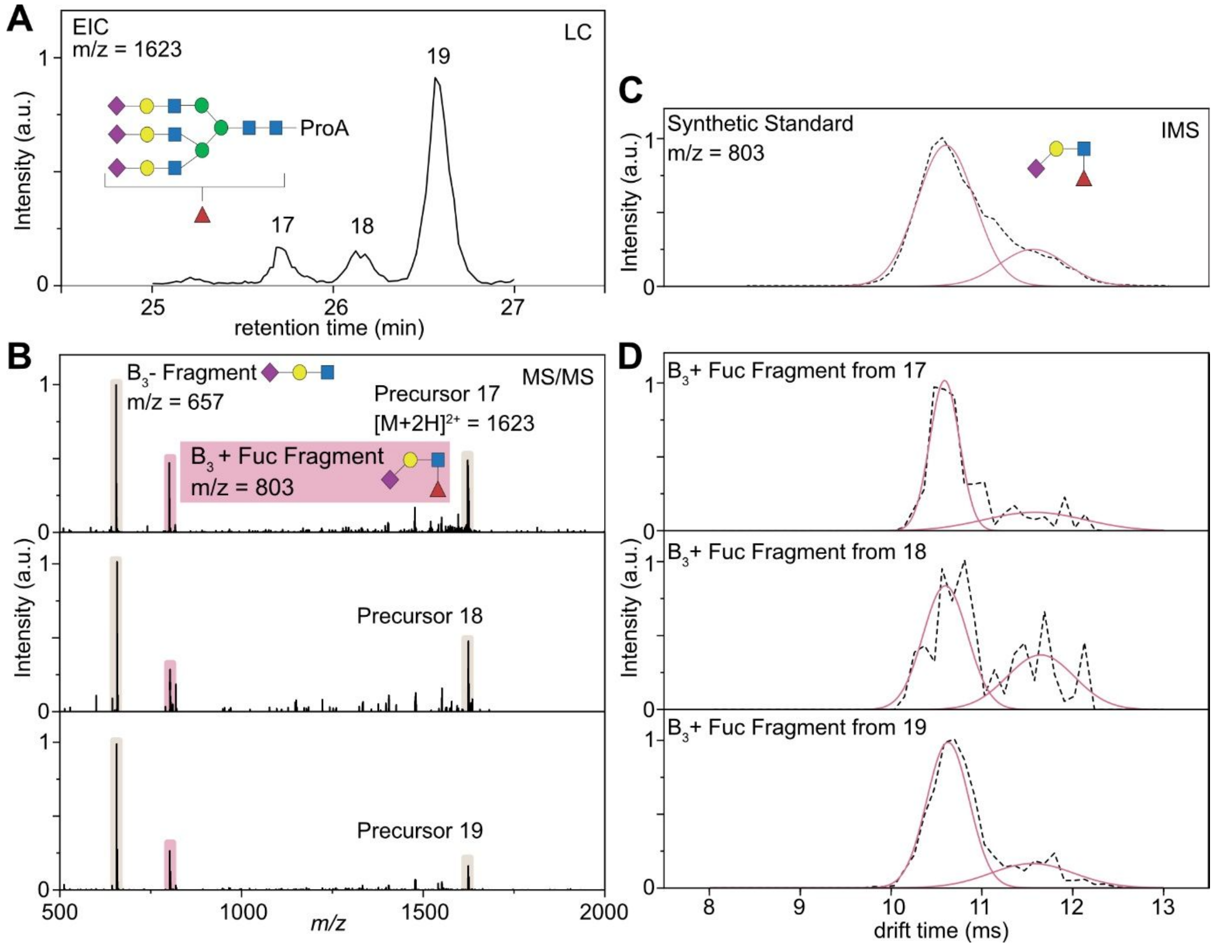

Figure S-1: Determination of fucosylation pattern for triantennary species. (A) Extracted ion chromatogram (EIC) of a triply sialylated triantennary glycan with one fucose attached (m/z = 1623). (B) MS/MS spectra of the precursor ions 17 to 19 which are almost identical and show the dominant B_3 trisaccharide fragment. In addition, all three species show the characteristic B_3 + fucose fragmentation (highlighted in red) which indicates antenna fucosylation. (C) Mobilogram of a synthetic standard (3´-Sialyl-Lewis-X). (d) Mobilograms of the B_3 + fucose fragment generated from all precursor ions. Comparison with the synthetic standard allows to identify the fucose isoforms and confirm the native state of the fucosylation. As the fucosylated species are generally low in intensity (especially in positive ion mode) the right shoulder of the ATD at ~11.6 ms exhibits a very low S/N ratio. The main signal at 10.5 ms, however, can still be used for unambigious assignment of the ATD.

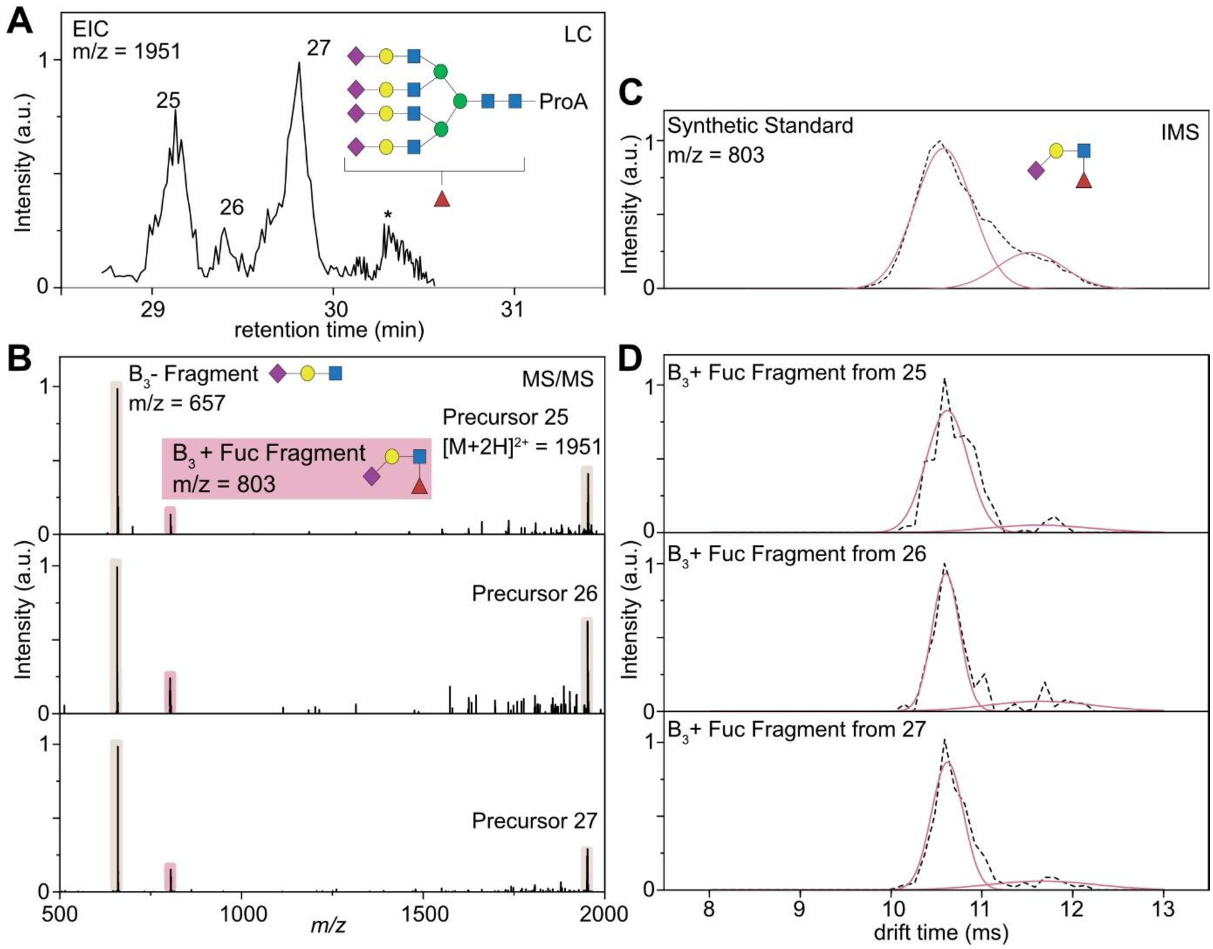

Figure S-2: Determination of fucosylation pattern for tetraantennary species. (A) Extracted ion chromatogram (EIC) of a quadruply sialylated tetraantennary glycan with one fucose attached (m/z = 1951). (B) MS/MS spectra of the precursor ions 25 to 27 which are almost identical and show the dominant B_3 trisaccharide fragment. In addition, all three species show the characteristic B_3 + fucose fragmentation (highlighted in red) which indicates antennary fucosylation. (C) Mobilogram of a synthetic standard (3´-Sialyl-Lewis-X). (D) Mobilograms of the B_3 + fucose fragment generated from all precursor ions. The intensity of the large tetraantennary species is very low in positive ion mode and therefore the fragment intensity is even lower. Therefore the shoulder on the right hand side of the ATD at ~11.6 ms is almost non-existent. The main signal at 10.5 ms, however, can still be used for unambigious assignment of the ATD.
